# Evaluation of the Molecular Landscape in PD-L1 Positive Metastatic NSCLC: Data from Campania, Italy

**DOI:** 10.3390/ijms23158541

**Published:** 2022-08-01

**Authors:** Pasquale Pisapia, Antonino Iaccarino, Caterina De Luca, Gennaro Acanfora, Claudio Bellevicine, Roberto Bianco, Bruno Daniele, Luisa Ciampi, Marco De Felice, Teresa Fabozzi, Luigi Formisano, Pasqualina Giordano, Cesare Gridelli, Giovanni Pietro Ianniello, Annamaria Libroia, Paolo Maione, Mariantonia Nacchio, Fabio Pagni, Giovanna Palmieri, Francesco Pepe, Gianluca Russo, Maria Salatiello, Antonio Santaniello, Rachele Scamarcio, Davide Seminati, Michele Troia, Giancarlo Troncone, Elena Vigliar, Umberto Malapelle

**Affiliations:** 1 Department of Public Health, University of Naples Federico II, 80131 Naples, Italy; pasquale.pisapia@unina.it (P.P.); antiaccc@hotmail.com (A.I.); caterina.deluca@unina.it (C.D.L.); gennaro.acanfora95@gmail.com (G.A.); claudio.bellevicine@unina.it (C.B.); mariantonia.nacchio@unina.it (M.N.); francesco.pepe4@unina.it (F.P.); gianlucar93@libero.it (G.R.); maria.salatiello@unina.it (M.S.); elena.vigliar@unina.it (E.V.); umberto.malapelle@unina.it (U.M.); 2 Department of Clinical Medicine and Surgery, University of Naples Federico II, 80131 Naples, Italy; roberto.bianco@unina.it (R.B.); luigi.formisano1@unina.it (L.F.); santaniellanto@gmail.com (A.S.); 3 Oncology Unit, Ospedale del Mare, 80147 Naples, Italy; b.daniele@libero.it (B.D.); fabozzit79@gmail.com (T.F.); giopas@email.it (P.G.); 4 Department of Pathology, Ente Ecclesiastico Ospedale Generale Regionale F. Miulli, 70021 Acquaviva delle Fonti, Italy; l.ciampi@miulli.it (L.C.); g.palmieri@miulli.it (G.P.); r.scamarcio@miulli.it (R.S.); m.troia@miulli.it (M.T.); 5 Department of Oncology, A.O.R.N. Sant’Anna e San Sebastiano, 81100 Caserta, Italy; mrc.defelice@gmail.com (M.D.F.); ianniellog@libero.it (G.P.I.); 6 Division of Medical Oncology, “S.G. Moscati” Hospital, 83100 Avellino, Italy; cgridelli@libero.it (C.G.); pmaione@libero.it (P.M.); 7 Oncology Unit, “Andrea Tortora” Hospital, ASL Salerno, 84016 Pagani, Italy; doctannalibroia@virgilio.it; 8 Department of Medicine and Surgery, Pathology, University of Milano-Bicocca, 20900 Monza, Italy; fabio.pagni@unimib.it (F.P.); d.seminati@campus.unimib.it (D.S.)

**Keywords:** molecular oncology, molecular pathology, PD-L1, immune-checkpoint inhibitors, biomarkers

## Abstract

Background: Immune-checkpoint inhibitors (ICIs) have increased and improved the treatment options for patients with non-oncogene-addicted advanced stage non-small cell lung cancer (NSCLC). However, the role of ICIs in oncogene-addicted advanced stage NSCLC patients is still debated. In this study, in an attempt to fill in the informational gap on the effect of ICIs on other driver mutations, we set out to provide a molecular landscape of clinically relevant oncogenic drivers in programmed death-ligand 1 (PD-L1) positive NSCLC patients. Methods: We retrospectively reviewed data on 167 advanced stage NSCLC PD-L1 positive patients (≥1%) who were referred to our clinic for molecular evaluation of five driver oncogenes, namely, *EGFR*, *KRAS*, *BRAF*, *ALK* and *ROS1*. Results: Interestingly, *n* = 93 (55.7%) patients showed at least one genomic alteration within the tested genes. Furthermore, analyzing a subset of patients with PD-L1 tumor proportion score (TPS) ≥ 50% and concomitant gene alterations (*n* = 8), we found that *n* = 3 (37.5%) of these patients feature clinical benefit with ICIs administration, despite the presence of a concomitant *KRAS* gene alteration. Conclusions: In this study, we provide a molecular landscape of clinically relevant biomarkers in NSCLC PD-L1 positive patients, along with data evidencing the clinical benefit of ICIs in patient NSCLC PD-L1 positive alterations.

## 1. Introduction

Lung cancer represents the leading cause of cancer deaths worldwide [1]. About 85% of lung cancers are non-small cell lung cancer (NSCLC) [2,3]. In recent years, several efforts have been made to improve clinical outcomes of advanced stage NSCLC patients. Central to these efforts has been the advent of precision medicine. This approach, which involves the identification of actionable oncogenic driver alterations, has spurred the development of specific therapeutic agents capable of thwarting the molecular pathways involved in cancer progression. Among these agents are tyrosine kinase inhibitors (TKIs). Remarkably, these agents are able to target a long series of recently discovered oncogenetic mutations involved in several driver genes, namely, Epidermal Growth Factor Receptor (*EGFR*) [4,5,6,7], V-Raf Murine Sarcoma Viral Oncogene Homolog B1 (*BRAF*) [8,9], Kirsten Rat Sarcoma Viral Oncogene Homolog (*KRAS*) exon 2 p.G12C [10,11] and gene fusions in Anaplastic Lymphoma Receptor Tyrosine Kinase (*ALK*) [12,13,14,15,16] and ROS Proto-Oncogene 1, Receptor Tyrosine Kinase (*ROS1*) [17,18,19]. Another milestone in the clinical management of advanced stage NSCLC patients has been the development of immune-checkpoint inhibitors (ICIs) [20]. Currently, the evaluation of programmed death-ligand 1 (PD-L1) expression levels is the most widely adopted and standardized tool for ICI administration [21,22]. ICIs have indeed increased and improved the treatment options for non-oncogene-addicted advanced stage NSCLC patients [23,24,25,26]. However, the role of ICIs in oncogene-addicted advanced stage NSCLC patients is still debated [27]. For example, a recent review has highlighted the lack of efficacy of pembrolizumab in naïve *EGFR*-mutated advanced stage NSCLC patients expressing low levels of PD-L1 (1%) [28]. However, even less is known about the effect of ICIs on other clinically relevant biomarkers. Undoubtedly, paucity of data in this specific field is a major setback for lung cancer treatment. Indeed, evaluating PD-L1 expression levels and the genomic assessment of clinically relevant oncogenic targetable drivers would be crucial to broaden the treatment options for NSCLC patients. In our referral laboratory experience at the Molecular Predictive Pathology Laboratory at the Department of Public Health of the University of Naples Federico II, we routinely perform immunohistochemistry/immunocytochemistry (IHC/ICC) to evaluate PD-L1 expression [29,30]. In addition, we perform both DNA-based next generation sequencing (NGS) and fully automated real-time polymerase chain reaction (RT-qPCR), namely, Idylla™ (Biocartis, Mechelen, Belgium) to evaluate point mutations, deletions and insertions [31,32,33] and IHC/ICC and RNA-based NGS analysis to identify gene fusions [34].

In this study, in an attempt to fill in the informational gap on the effect of ICIs on other driver mutations, we set out to provide a molecular landscape of clinically relevant oncogenic drivers in PD-L1 positive NSCLC patients. To this aim, we retrospectively evaluated data collected from our archives of advanced stage NSCLC patients with positive PD-L1 expression (≥1%) who were referred to our clinic for evaluation of at least five of the most common driver mutations, namely, *EGFR*, *KRAS*, *BRAF*, *ALK* and *ROS1*. In addition, in a subset of patients, we were also able to retrieve information about patients’ medical treatments and performance status.

## 2. Results

### 2.1. Patient and Sample Characteristics

We retrospectively analyzed data on a total of 167 samples from advanced stage NSCLC PD-L1 positive patients (≥1%) who were referred to our clinic for molecular evaluation of at least five proto-oncogenes, namely, *EGFR*, *KRAS*, *BRAF*, *ALK* and *ROS1*. Overall, our study population was composed of *n* = 103 (61.7%) males and *n* = 64 (38.3%) females with a median age of 67.3 years (ranging from 43 to 93 years). The vast majority was diagnosed with adenocarcinoma (ADC) (*n* = 62, 37.1%), NSCLC favor ADC (*n* = 58, 34.7% and NSCLC not otherwise specified (NOS) (*n* = 32, 19.2%), followed by squamous cell carcinoma (SqCC) (*n* = 8, 4.8%), NSCLC favor SqCC (*n* = 4, 2.4%) and adeno-squamous carcinoma (*n* = 3, 1.8%). The number of histological samples (*n* = 110, 65.9%) was almost double that of cytological samples (*n* = 57, 34.1%). Histological samples comprised small biopsies (*n* = 86, 78.2%), and surgical resections (*n* = 24, 21.8%). As for the cytological samples, they were mostly made up of cell blocks (*n* = 52, 91.2%); of these, some were used for PD-L1 TPS assessment. Direct smears (*n* = 5, 8.8%), instead, were used for the assessment of other clinically relevant biomarkers.

**Figure 1:**
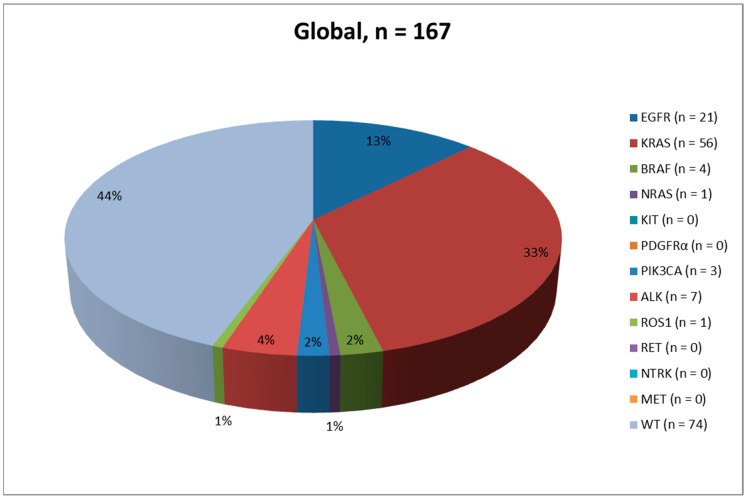
Pie chart describing the mutational landscape in the global advanced stage NSCLC PD-L1 positive patients (≥1%) population.

**Figure 2:**
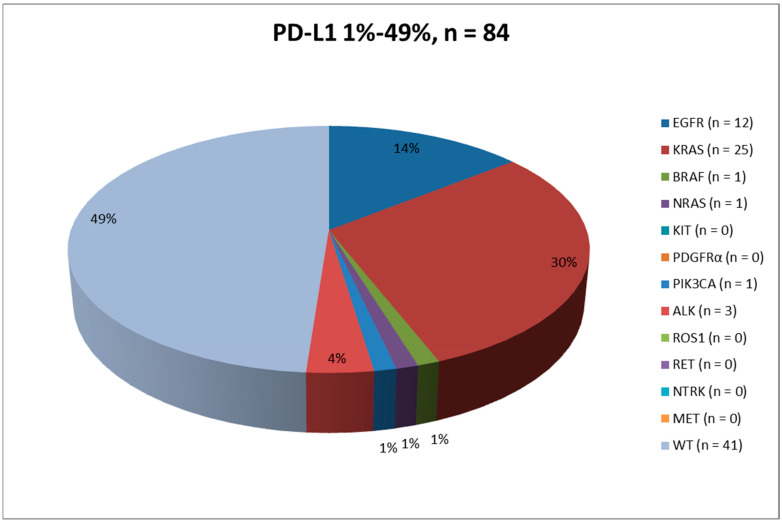
Pie chart describing the mutational landscape in the 1–49% PD-L1 positive advanced stage NSCLC patients population.

**Figure 3:**
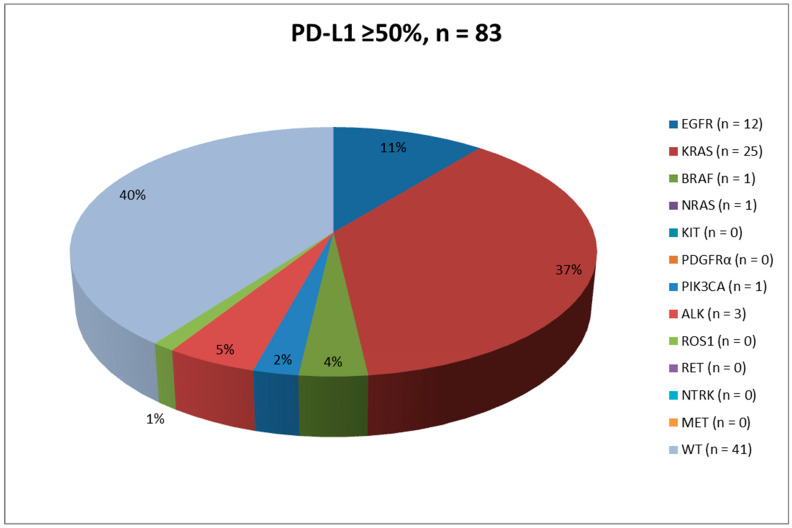
Pie chart describing the mutational landscape in the ≥50% PD-L1 positive advanced stage NSCLC patients population.

Results are summarized in Table 1, Figure 1, Figure 2 and Figure 3 and Supplementary Table S1.

### 2.2. PD-L1 Status and Molecular Evaluation

**Figure 4:**
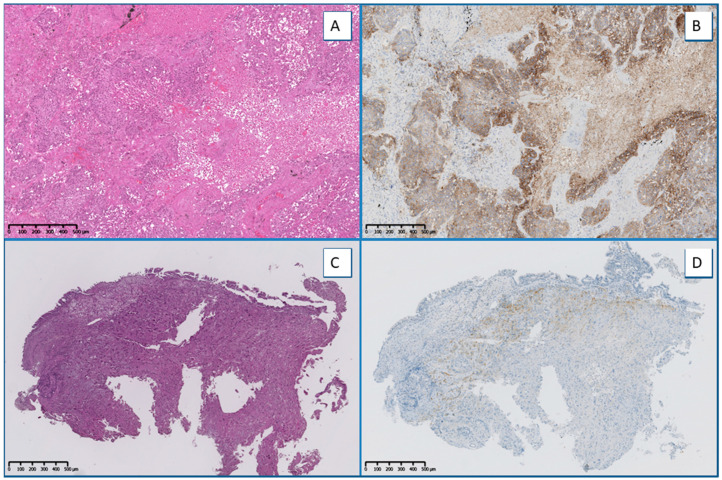
Exemplificative cases of PD-L1 SP263 clone IHC evaluation. Original magnification 5×: (**A**) H and E stained slide with the corresponding PD-L1 evaluation (1–49%, (**B**)); (**C**) H and E stained slide with the corresponding PD-L1 evaluation (≥50%, (**D**)).

For the evaluation of the expression level of PD-L1, SP263 (*n* = 134, 80.2%) was more commonly used than 22C3 (*n* = 33, 19.8%). Overall, *n* = 84 (50.3%) samples expressed PD-L1 levels between 1% and 49%, and *n* = 83 (49.7%) samples expressed PD-L1 levels ≥50% (Figure 4). For the evaluation of DNA-based biomarkers, NGS was used to analyze 164/167 (98.2%) cases, whereas RT-qPCR analysis was used for the remaining 3 cases (1.8%). Remarkably, *KRAS* was the most commonly mutated gene (*n* = 56, 33.5%), followed by *EGFR* (*n* = 21, 12.6%), *BRAF* (*n* = 4, 2.4%), Phosphatidylinositol-4,5-Bisphosphate 3-Kinase Catalytic Subunit Alpha (*PIK3CA*, *n* = 3, 1.8%), and Neuroblastoma RAS Viral Oncogene Homolog (*NRAS*, *n* = 1, 0.6%). No alterations were reported in KIT Proto-Oncogene, Receptor Tyrosine Kinase (*KIT*) and Platelet Derived Growth Factor Receptor Alpha (*PDGFRα*). For the evaluation of *ALK* and *ROS1* gene rearrangements, IHC/ICC was employed in the vast majority of cases (*n* = 152, 91.0%), whereas RNA-based NGS analysis was adopted in only *n* = 15 (9.0%) instances. Interestingly, *ALK* fusions emerged in *n* = 7 (4.2%) cases and *ROS1* in only *n* = 1 (0.6%) case. No additional Rearranged During Transfection (*RET*) and Neurotrophic Receptor Tyrosine Kinase (*NTRK*) gene fusions or MET Proto-Oncogene and Receptor Tyrosine Kinase (*MET*) exon 14 skipping alterations were reported. As for the biomarker analyses, *n* = 93 (55.7%) cases showed at least one genomic alteration within the tested genes, whereas no concomitant clinically relevant biomarker alterations were detected in the remaining *n* = 74 (44.3%) cases.

Results are summarized in Table 1, Figure 1, Figure 2, Figure 3 and Figure 4 and Supplementary Table S1.

### 2.3. PD-L1 Expression: 1–49%

For DNA-based biomarker analyses, NGS was applied to almost all cases (*n* = 83, 98.8%), whereas RT-qPCR was used for only *n* = 1 (1.2%) case. Remarkably, *KRAS* was the most commonly mutated gene (*n* = 25, 29.8%), followed by *EGFR* (*n* = 12, 14.3%), *BRAF* (*n* = 1, 1.2%), *PIK3CA* (*n* = 1, 1.2%) and *NRAS* (*n* = 1, 1.2%). Regarding the evaluation of *ALK* and *ROS1* gene rearrangements, IHC/ICC was employed in the vast majority of cases (*n* = 78, 92.9%), whereas RNA-based NGS analysis was adopted in only *n* = 6 (7.1%) instances. Interestingly, *n* = 3 (3.6%) cases showed *ALK* gene fusion, whereas no *ROS1* gene rearrangements were reported. Concerning the biomarker analyses, *n* = 43 (51.2%) cases showed at least one genomic alteration within the tested genes, whereas no concomitant clinically relevant biomarker alterations were detected in the remaining *n* = 41 (48.8%) cases.

Results are summarized in Table 1, Figure 1, Figure 2 and Figure 3 and Supplementary Table S1.

### 2.4. PD-L1 Expression: ≥50%

For DNA-based biomarker analyses, NGS was applied to almost all cases (*n* = 81, 97.6%), whereas RT-qPCR was employed in only *n* = 2 (2.4%) instances. Remarkably, *KRAS* was the most frequently mutated gene (*n* = 31, 37.3%), followed by *EGFR* (*n* = 9, 10.8%), *BRAF* (*n* = 3, 3.6%) and *PIK3CA* (*n* = 2, 2.5%), No alterations were reported in *NRAS*. Regarding the evaluation of *ALK* and *ROS1* gene rearrangements, IHC/ICC was employed in the vast majority of cases (*n* = 74, 89.2%), whereas RNA-based NGS analysis was adopted in only *n* = 9 (10.8%) instances. Interestingly, whereas *ALK* fusions were identified in *n* = 4 (4.8%) cases, *ROS1* fusions were detected in only *n* = 1 (1.2%) case. As for the biomarker analyses, at least one genomic alteration was detected in *n* = 50 (60.2%) cases, whereas no concomitant clinically relevant biomarker alterations were detected in the remaining *n* = 33 (39.8%) cases.

Results are summarized in Table 1, Figure 1, Figure 2 and Figure 3 and Supplementary Table S1.

### 2.5. Clinical Management

Overall, data on the clinical management of *n* = 41 patients were retrieved. Among these, *n* = 16 (39.0%) showed a PD-L1 expression ≥50%. Half of these patients did not show concomitant gene alterations. In this subset, *n* = 6 (75.0%) patients received immunotherapy alone, *n* = 1 patient chemotherapy alone (1/8, 12.5%), and *n* = 1 patient supportive care (1/8, 12.5%). Interestingly, five out of six patients (83.3%) are still receiving frontline treatments comprising ICIs alone or combination therapies. In the abovementioned subset of patients with concomitant gene alterations, almost all patients (7/8, 87.5%) presented with *KRAS* gene mutations (*n* = 4 *KRAS* exon 2 p.G12C, *n* = 1 *KRAS* exon 2 p.G12A, *n* = 1 *KRAS* exon 2 p.G12V and *n* = 1 *KRAS* exon 3 p.Q61H), whereas one patient harbored one type of *EML4*(6)-*ALK*(20) gene rearrangement. Seven cases harboring *KRAS* gene mutations were treated with pembrolizumab. Overall, *n* = 3 (37.5%) of these patients (*n* = 2 with a *KRAS* exon 2 p.G12C and *n* = 1 with *KRAS* exon 2 p.G12A) are still being treated with the same therapeutic regimen.

Results are summarized in Table 2.

## 3. Discussion

The evaluation of PD-L1 expression is now one of the mandatory predictive tests to conduct in advanced stage NSCLC patients. In this study, we retrospectively analyzed a total of 167 advanced stage NSCLC PD-L1 positive patients (≥1%) who were referred to our referral clinic for the molecular evaluation of at least five driver genes, namely, *EGFR*, *KRAS*, *BRAF*, *ALK* and *ROS1*. In our experience, both histological (*n* = 110, 65.9%) and cytological (*n* = 57, 34.1%) samples were analyzed, strongly supporting the evidence that evaluation of PD-L1 expression levels and molecular profiling of advanced stage NSCLC patients is feasible by using both types of specimens [21,22,29,30]. In this context, studies have shown that NGS (both DNA- and RNA-based approaches) represents a valid solution to analyze clinically relevant biomarkers simultaneously in small tissue samples [32,33]. Overall, *n* = 84 (50.3%) and *n* = 83 (49.7%) patients showed a PD-L1 expression level of 1–49% and ≥50%, respectively. As in other experiences, most of the patients (*n* = 103, 61.7%; *n* = 53, 63.1%; *n* = 50, 60.2% were males [35]. Most cases were diagnosed as ADC (*n* = 62, 37.1%; *n* = 41, 48.8%; *n* = 21, 25.3%), NSCLC favor ADC (*n* = 58, 34.7%; *n* = 24, 28.6%; *n* = 34, 41.0%), and NSCLC NOS (*n* = 32, 19.2%; *n* = 11, 13.1%; *n* = 21, 25.3%), followed by SqCC (*n* = 8, 4.8%; *n* = 6, 7.1%; *n* = 2, 2.4%), NSCLC favor SqCC (*n* = 4, 2.4%; *n* = 1, 1.2%; *n* = 3, 3.6%) and adeno-squamous carcinomas (*n* = 3, 1.8%; *n* = 1, 1.2%; *n* = 2, 2.4%). Interestingly, *n* = 93 (55.7%) patients showed at least one genomic alteration within the tested genes. From an epidemiological point of view, the most common genomic alterations were reported within the *KRAS* gene (*n* = 56, 33.5%), followed by *EGFR* (*n* = 21, 12.6%), *ALK* (*n* = 7, 4.2%), *BRAF* (*n* = 4, 2.4%), *PIK3CA* (*n* = 3, 1.8%), *ROS1* (*n* = 1, 0.6%), and *NRAS* (*n* = 1, 0.6%) (Table 1 and Supplementary Table S1). The strong correlation between PD-L1 expression and *KRAS* mutations has been previously demonstrated. Karatrasoglou et al. highlighted that PD-L1 positive (TPS ≥ 1%) NSCLC patients showed a concomitant *KRAS* mutation, and in particular *KRAS* exon 2 p,G12C point mutation, in a higher percentage of patients with respect to PD-L1 negative patients [36]. This phenomenon may be related to the induction of PD-L1 by *KRAS* mutations as it has been demonstrated in human NSCLC cell lines [37,38,39].

As for the data on treatment regimens, the seven cases harboring *KRAS* gene mutations received pembrolizumab alone (6/7, 85.7%) or pembrolizumab plus carboplatin and pemetrexed (1/7, 14.3%). Overall, in *n* = 3 (37.5%) of these patients (*n* = 2 with a *KRAS* exon 2 p.G12C and *n* = 1 with *KRAS* exon 2 p.G12A) the treatment is still ongoing. These data support the role of *KRAS* mutations (in particular *KRAS* exon 2 p.G12C point mutation) in increasing ICI sensitivity [40].

In this setting, despite the role of ICIs has been clearly demonstrated in the treatment of high PD-L1 expressers [23], little is known about the role of concomitant genomic alterations on this regimen. Lee et al. showed that ICI administration in *KRAS* mutated patients may determine an overall survival (OS) benefit respect to *KRAS* wild-type patients [41]. Similarly, Bodor et al. highlighted that *KRAS*-mutated NSCLC patients with PD-L1 TPS≥1% had a longer progression-free survival respect to PD-L1 negative patients (4.1 vs. 3.2 months, *p*  =  0.001) [42]. A possible explanation may be the presence of a specific interaction between the tumor microenvironment and ICIs for this specific subset of patients as demonstrated by Falk et al. [43]. Similarly, the adoption of front-line pembrolizumab in PD-L1 positive advanced stage NSCLC patients harboring a *KRAS* exon 2 p.G12C point mutation seemed to be predictive of higher objective response rate (ORR, 57% versus 29%), median progression free survival (PFS, 12 versus 6 months) and OS (28 versus 15 months) [44]. Different from *KRAS* exon 2 p.G12C, the identification of other concomitant driver mutations is predictive of poor response to ICIs administration in the PD-L1 positive population [27]. The limited efficacy of ICIs in patients harboring *EGFR* mutations has been widely demonstrated [45]. In a phase II study, Lisberg et al. highlighted the absence of response to pembrolizumab as first line approach in advanced stage PD-L1 positive *EGFR*-mutant NSCLC patients naïve to TKI administration [28]. Similar data have been reported for other ICI drugs, such as atezolizumab and durvalumab [46,47]. The role of ICIs is controversial in *BRAF*-mutated patients [48]. In fact, in a multicentric retrospective cohort, Dudnik et al. showed promising data in terms of clinical efficacy of ICIs in *BRAF*-mutated advanced stage NSCLC [49]. Conversely, in a small retrospective study, Tan et al. highlighted an inferior OS in *BRAF*-mutated patients receiving ICI respect to those treated with front-line chemotherapy [50]. Regarding gene rearrangements, a very limited efficacy of ICIs in *ALK-* [47,51,52,53,54], *ROS1-* [55,56], *RET-* [57] and *NTRK*-rearranged [27] NSCLC patients has been highlighted. Considering *MET* exon 14 skipping, despite some evidence reporting response to ICIs [58], the overall efficacy of immunotherapy respect to target therapy is quite modest [59].

In conclusion, in this study we have provided a real-world practice experience on the molecular landscape of clinically relevant biomarkers in NSCLC PD-L1-positive patients. The most significant limitations of our study were the limited number of cases, the absence of molecular data on PD-L1 negative patients, the limited number of gene alterations analyzed and clinical data on progression-free survival and overall survival and the lack of clinical data on the vast majority of patients. Further studies are thus needed to better assess the role of the complex genomic landscape in advanced stage NSCLC patients.

## 4. Materials and Methods

### 4.1. Study Design

**Figure 5:**
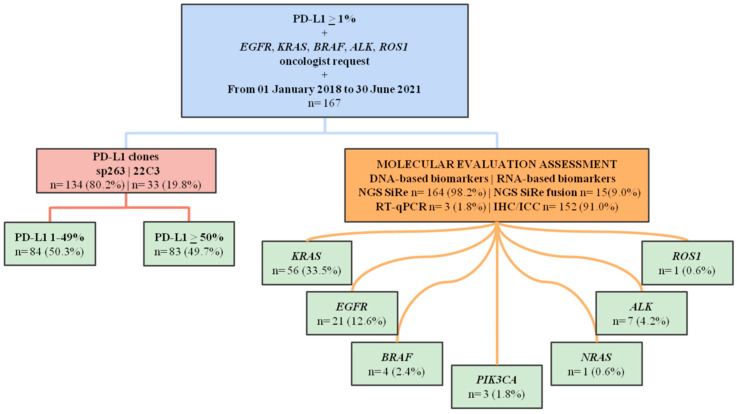
Study design and results. Abbreviations: *ALK*: Anaplastic Lymphoma Receptor Tyrosine Kinase; *BRAF*: V-Raf Murine Sarcoma Viral Oncogene Homolog B1; *EGFR*: Epidermal Growth Factor Receptor; ICC: immunocytochemistry; IHC: immunohistochemistry; *KRAS*: Kirsten Rat Sarcoma Viral Oncogene Homolog; NGS: next generation sequencing; *NRAS*: Neuroblastoma RAS Viral Oncogene Homolog; PD-L1: programmed death-ligand 1; *PIK3CA*: Phosphatidylinositol-4,5-Bisphosphate 3-Kinase Catalytic Subunit Alpha; RT-qPCR: real-time polymerase chain reaction; *ROS1*: ROS Proto-Oncogene 1, Receptor Tyrosine Kinase.

In this study, we retrospectively reviewed cases referred to our clinic from 1 January 2018 to 30 June 2021 for molecular evaluation of at least five driver druggable oncogenes, namely, *EGFR*, *KRAS*, *BRAF*, *ALK*, *ROS1* and PD-L1 expression assessment; PD-L1 positive cases (expression in ≥1% tumor cells) were selected. Information regarding sex, median age, sample type and subtype and diagnosis was also retrieved. (Figure 5) Furthermore, for a subset of patients, data related to the duration of the first-line treatment, or until the loss of data for any causes, were also gathered.

All information regarding human material was managed using anonymous numerical codes, and all samples were handled in compliance with the Declaration of Helsinki (http://www.wma.net/en/30publications/10policies/b3/, last accessed 30 June 2022).

### 4.2. IHC/ICC Analysis

PD-L1 IHC/ICC evaluation was performed with a validated laboratory developed test (LDT), consisting of the use of Dako’s concentrate 22C3 anti-PD-L1 primary antibody with a Ventana’s detection systems on the BenchMark XT platform, or by using the companion diagnostic kit SP263 assay (Ventana Medical Systems, Tucson, AZ, USA) [29,30]. The level of PD-L1 expression was determined by using tumor proportion score (TPS). PD-L1 positive cases were classified either as low-positive PD-L1 expression (1–49%) or as high-positive PD-L1 expression (≥50%) [29,30].

ALK IHC/ICC evaluation was performed by using the Ventana ALK D5F3 companion diagnostic (CDx) assay (Ventana Medical Systems) together with the OptiView (Ventana) detection system. The latter system features a tyramide-based amplification phase in addition to the polymeric step. In particular, by increasing the signal difference between the specific immunoreaction of neoplastic cells and the background, the amplification phase significantly reduces equivocal results. Thus, only positive or negative ALK cases can be reported. Typically, only strong and granular cytoplasmic signals are scored as positive, regardless of the percentage of stained neoplastic cells [60,61,62].

ROS1 IHC/ICC evaluation was carried out with the D4D6 (Cell Signaling Technology, Inc., Danvers, MA, USA) clone. Generally, only tumors with a moderate- to strong staining intensity signal (2+ or 3+ scores) in more than half of the neoplastic cells are considered positive [60,63,64].

Finally, ALK and ROS1 IHC/ICC assays were adopted to confirm RNA-based NGS positive cases.

### 4.3. Molecular Testing

DNA- and RNA- based analyses of samples were carried out. DNA-based NGS analysis was performed with our narrow NGS panel, namely, SiRe^®^ [65]; this panel was designed, developed and validated in the Molecular Predictive Pathology Laboratory of the Department of Public Health at the University of Naples Federico II [65]. SiRe^®^ can simultaneously detect multiple hotspot gene alterations in seven genes (*EGFR*, *KRAS*, *BRAF*, *NRAS*, *KIT*, *PDGFRα*, and *PIK3CA*) [31,65]. In the present study, only variants with allele coverage >20X and a quality score >20, with an amplicon coverage of at least 500X alleles, were called.

RNA-based NGS analysis was performed with a narrow NGS panel, namely, SiRe fusion [34]. This panel was also designed, developed, and validated in the Molecular Predictive Pathology Laboratory of the Department of Public Health at University of Naples Federico II [34]. It simultaneously detects alterations in six oncogenic genes, namely, *ALK*, *ROS1*, *RET*, *NTRK* gene rearrangements, *MET* exon 14 skipping alterations [34]. In all the study cases, *ALK* and *ROS1* status was further confirmed with IHC/ICC.

In a limited number of cases, the fully automated Idylla™ RT-qPCR platform was adopted to evaluate the molecular status of *EGFR*, *KRAS* and *BRAF* [32,33,66].

## Figures and Tables

**Table 1:** Clinical and molecular findings of the study population.

	Global	1–49%	≥50%
Total (%)	167 (100.0)	84 (100.0)	83 (100.0)
Sex (%)	M: 103 (61.7) F: 64 (38.3)	M: 53 (63.1) F: 31 (36.9)	M: 50 (60.2) F: 33 (39.8)
Median Age (range)	67.3 y (43–93 y)	66.9 y (43–92 y)	67.8 y (44–93 y)
Sample type (*n*; %) - subtype (*n*; %)	Histological (110, 65.9) - Biopsy (86, 78.2) - Resection (24, 21.8) Cytological (57, 34.1) - Cell block (52, 91.2) - Smear (5, 8.8)	Histological (53, 63.1) - Biopsy (40, 75.5) - Resection (13, 24.5) Cytological (31, 36.9) - Cell block (29, 93.5) - Smear (2, 6.5)	Histological (57, 68.7) - Biopsy (46, 80.7) - Resection (11, 19.3) Cytological (26, 31.3) - Cell block (23, 88.5) - Smear (3, 11.5)
Diagnosis (*n*, %)	ADC (62, 37.1) NSCLC favor ADC (58, 34.7) NSCLC NOS (32, 19.2) SqCC (8, 4.8) NSCLC favor SqCC (4, 2.4) ADC + SqCC (3, 1.8)	ADC (41, 48.8) NSCLC favor ADC (24, 28.6) NSCLC NOS (11, 13.1) SqCC (6, 7.1) NSCLC favor SqCC (1, 1.2) ADC + SqCC (1, 1.2)	NSCLC favor ADC (34, 41.0) ADC (21, 25.3) NSCLC NOS (21, 25.3) NSCLC favor SqCC (3, 3.6) SqCC (2, 2.4) ADC + SqCC (2, 2.4)
PD-L1 (*n*, %)	1–49 (84, 50.3) ≥50 (83, 49.7)	-	-
Clone (*n*, %)	SP263 (134, 80.2) 22C3 (33, 19.8)	SP263 (67, 79.8) 22C3 (17, 20.2)	SP263 (67, 80.7) 22C3 (16, 19.3)
DNA based-biomarker molecular platform (*n*, %)	NGS (164, 98.2) RT-qPCR (3, 1.8)	NGS (83, 98.8) RT-qPCR (1, 1.2)	NGS (81, 97.6) RT-qPCR (2, 2.4)
Molecular results (*n*, %)	WT (74, 44.3) Mutated (93, 55.7)	WT (41, 48.8) Mutated (43, 51.2)	WT (33, 39.8) Mutated (50, 60.2)
DNA-based biomarkers (*n*, %)	*EGFR* (167, 100.0) - WT (146, 87.4) - mutated (21, 12.6) - p.L858R (9, 42.8) - p.E746_A750del (6, 28.4) - p.E709_T710insD (1, 4.8) - p.G719A + p.T790M (1, 4.8) - p.I744_K745insKIPVAI (1, 4.8) - p.E746_S752del (1, 4.8) - p.S768_D760dup (1, 4.8) - p.S768I (1, 4.8) *KRAS* (167, 100.0) - WT (111, 66.5) - mutated (56, 33.5) - p.G12C (27, 48.1) - p.G12V (13, 23.2) - p.G12A (5, 8.9) - p.G12D (3, 5.4) - p.Q61H (3, 5.4) - p.G13C (2, 3.6) - p.G12R (1, 1.8) - p.G13D (1, 1.8) - p.G13R (1, 1.8) *BRAF* (167, 100.0) - WT (163, 97.6) - mutated (4, 2.4) - p.V600E (2, 50.0) - p.G466V (1, 25.0) - p.G469A (1, 25.0) *NRAS* (164, 98.2) - WT (163, 99.4) - mutated (1, 0.6) - p.G12D (1, 100.0) *KIT* (164, 98.2) - WT (164, 100.0) *PDGFRα* (164, 98.2) - WT (164, 100.0) *PIK3CA* (164, 98.2) - WT (161, 98.2) - mutated (3, 1.8) - p.E545K (2, 66.7) - p.E542K (1, 33.3)	*EGFR* (84, 100.0) - WT (72, 85.7) - mutated (12, 14.3) - p.E746_A750del (4, 33.4) - p.L858R (4, 33.4) - p.E709_T710indD (1, 8.3) - p.G719 + p.T790M (1, 8.3) - p.S768_D760dup (1, 8.3) - p.S768I (1, 8.3) *KRAS* (84, 100.0) - WT (59, 70.2) - mutated (25, 29.8) - p.G12C (10, 40.0) - p.G12V (8, 32.0) - p.G13C (2, 8.0) - p.G12A (1, 4.0) - p.G12D (1, 4.0) - p.G12R (1, 4.0) - p.G13R (1, 4.0) - p.Q61H (1, 4.0) *BRAF* (84, 100.0) - WT (83, 98.8) - mutated (1, 1.2) - p.G469A (1, 100.0) *NRAS* (83, 98.8) - WT (82, 98.8) - mutated (1, 1.2) - p.G12D (1, 100.0) *KIT* (83, 98.8) - WT (83, 100.0) *PDGFRα* (83, 98.8) - WT (83, 100.0) *PIK3CA* (83, 98.8) - WT (82, 98.8) - mutated (1, 1.2) - p.E542K (1, 100.0)	*EGFR* (83, 100.0) - WT (74, 89.2) - mutated (9, 10.8) - p.L858R (5, 55.6) - p.E746_A750del (2, 22.2) - p.I744_K745insKIPVAI (1, 11.1) - p.E746_S752del (1, 11.1) *KRAS* (83, 100.0) - WT (52, 62.7) - mutated (31, 37.3) - p.G12C (17, 54.8) - p.G12V (5, 16.1) - p.G12A (4, 12.9) - p.G12D (2, 6.5) - p.Q61H (2, 6.5) - p.G13D (1, 3.2) *BRAF* (83, 100.0) - WT (80, 96.4) - mutated (3, 3.6) - p.V600E (2, 66.7) - p.G466V (1, 33.3) *NRAS* (81, 97.6) - WT (81, 100.0) *KIT* (81, 97.6) - WT (81, 100.0) *PDGFRα* (81, 97.6) - WT (81, 100.0) *PIK3CA* (81, 97.6) - WT (79, 97.5) - mutated (2, 2.5) - p.E545K (2, 100.0)
RNA-based biomarker assays (*n*, %)	IHC/ICC (152, 91.0) NGS (15, 9.0)	IHC/ICC (78, 92.9) NGS (6, 7.1)	IHC/ICC (74, 89.2) NGS (9, 10.8)
RNA-based biomarkers (*n*, %)	*ALK* (167, 100.0) - Negative/WT (160, 95.8) - Positive/rearranged (7, 4.2) *ROS1* (167, 100.0) - Negative/WT (166, 99.4) - Positive/rearranged (1, 0.6) *RET* (15, 9.0) - WT (15, 100.0) *NTRK* (15, 9.0) - WT (15, 100.0) *MET* (15, 9.0) - WT (15, 100.0)	*ALK* (84, 100.0) - Negative/WT (81, 96.4) - Positive/rearranged (3, 3.6) *ROS1* (84, 100.0) - Negative/WT (84, 100.0) *RET* (6, 7.1) - WT (6, 100.0) *NTRK* (6, 7.1) - WT (6, 100.0) *MET* (6, 7.1) - WT (6, 100.0)	*ALK* (83, 100.0) - Negative/WT (79, 95.2) - Positive/rearranged (4, 4.8) *ROS1* (83, 100.0) - Negative/WT (82, 98.8) - Positive/rearranged (1, 1.2) *RET* (9, 10.8) - WT (9, 100.0) *NTRK* (9, 10.8) - WT (9, 100.0) *MET* (9, 10.8) - WT (9, 100.0)

Abbreviations: ADC: adenocarcinoma; *ALK*: Anaplastic Lymphoma Receptor Tyrosine Kinase; *BRAF*: V-Raf Murine Sarcoma Viral Oncogene Homolog B1; *EGFR*: Epidermal Growth Factor Receptor; F: female; ICC: immunocytochemistry; IHC: immunohistochemistry; *KIT*: KIT Proto-Oncogene, Receptor Tyrosine Kinase; *KRAS*: Kirsten Rat Sarcoma Viral Oncogene Homolog; M: male; *MET*: MET Proto-Oncogene, Receptor Tyrosine Kinase; *n*: number; NGS: next generation sequencing; NOS: not otherwise specified; *NRAS*: Neuroblastoma RAS Viral Oncogene Homolog; NSCLC: non-small cell lung cancer; *NTRK*: Neurotrophic Receptor Tyrosine Kinase; PD-L1: programmed death-ligand 1; *PDGFRα*: Platelet Derived Growth Factor Receptor Alpha; *PIK3CA*: Phosphatidylinositol-4,5-Bisphosphate 3-Kinase Catalytic Subunit Alpha; *RET*: Rearranged During Transfection; RT-qPCR: real-time polymerase chain reaction; *ROS1*: ROS Proto-Oncogene 1, Receptor Tyrosine Kinase; SqCC: squamous cell carcinoma; WT: wild type; y: years.

**Table 2:** Clinical management.

Sex	Age	Sample Type	Sample Subtype	Site	Diagnosis	PD-L1	Clone	Alteration	First Oncological Observation Date	Performance Status	First Line Treatment	First-Line Treatment Starting Date	First-Line Treatment End Date
M	70	Histological	Biopsy	Brain	NSCLC favor ADC	≥50%	SP263	*KRAS* exon 2 p.G12C	March 2019	1	Pembrolizumab	May 2019	Ongoing
F	66	Histological	Resection	Brain	ADC	≥50%	SP263	WT	February 2020	1	Pembrolizumab	March 2021	December 2021
M	77	Histological	Biopsy	Lymphnode	NSCLC favor ADC	1–49%	SP263	*KRAS* exon 2 p.G12V	May 2020	1	Carboplatino + Pemetrexed + Pembrolizumab	June 2020	April 2021
M	75	Histological	Biopsy	Lymphnode	ADC	≥50%	SP263	WT	April 2020	1	Durvalumab	February 2021	Ongoing
F	75	Histological	Biopsy	Lung	ADC	1–49%	SP263	*KRAS* exon 2 p.G12C	April 2020	0	Carboplatino-Pemetrexed	July 2020	Ongoing
M	57	Histological	Biopsy	Lung	ADC-SqCC	≥50%	SP263	WT	June 2020	0	Pembrolizumab	July 2020	Ongoing
M	77	Histological	Resection	Lung	ADC	≥50%	SP263	*KRAS* exon 2 p.G12C	February 2020	0	Pembrolizumab	September 2021	March 2022
F	69	Histological	Biopsy	Lung	NSCLC favor ADC	1–49%	SP263	*BRAF* exon 11 p.G469A	June 2020	2	Carboplatino + Pemetrexed + Pembrolizumab	September 2020	February 2021
F	69	Histological	Biopsy	Lung	NSCLC favor ADC	≥50%	SP263	*EML4*(6)-*ALK*(20)	September 2020	2	Brigatinib	February 2021	June 2021
F	68	Histological	Resection	Lymphnode	ADC	1–49%	SP263	WT	April 2019	0	Carboplatino + Pemetrexed	April 2019	Ongoing with only pemetrexed
F	69	Cytological	Cell block	Soft tissue	ADC	1–49%	SP263	*EGFR* exon 20 p.S768_D760dup	December 2019	1	Carboplatino + Pemetrexed + Pembrolizumab	February 2020	September 2020
M	57	Histological	Biopsy	Lung	NSCLC-NOS	≥50%	SP263	*KRAS* exon 2 p.G12C	February 2020	2	Pembrolizumab	March 2020	March 2020
F	55	Cytological	Cell block	Lung	NSCLC-NOS	≥50%	SP263	WT	December 2020	1	Pembrolizumab	January 2021	Ongoing
M	62	Histological	Biopsy	Pleura	ADC	1–49%	SP263	ALK positive	April 2021	1	Alectinib	April 2021	Ongoing
M	72	Cytological	Cell block	Lung	NSCLC favor ADC	1–49%	22C3	WT	June 2018	2	Carboplatin + Pemetrexed	July 2018	September 2018
M	78	Cytological	Smear	Lung	ADC	≥50%	SP263	WT	May 2019	2	Carboplatin	June 2019	September 2019
M	72	Cytological	Cell block	Lung	NSCLC favor ADC	1–49%	SP263	*KRAS* exon 2 p.G12C	March 2019	1	Carboplatin + Pemetrexed	March 2019	January 2020
F	73	Histological	Biopsy	Lung	NSCLC favor ADC	≥50%	SP263	*KRAS* exon 2 p.G12V	June 2019	0	Pembrolizumab	June 2019	January 2021
M	79	Histological	Biopsy	Lung	ADC	1–49%	SP263	WT	September 2019	0	Carboplatin + Pemetrexed	October 2019	January 2020
M	49	Cytological	Cell block	Lung	NSCLC favor ADC	≥50%	SP263	WT	December 2019	0	Pembrolizumab	December 2019	Ongoing
M	60	Histological	Biopsy	Lung	NSCLC favor ADC	≥50%	SP263	*KRAS* exon 3 p.Q61H	December 2019	0	Pembrolizumab	January 2020	April 2020
F	62	Histological	Biopsy	Brain	NSCLC-NOS	1–49%	SP263	*EGFR* exon 19 p.E746_A750del	December 2020	0	Osimertinib	January 2021	Ongoing
F	58	Histological	Resection	Brain	ADC	1–49%	SP263	WT	March 2021	0	Carboplatin + Pemetrexed + Pembrolizumab	April 2021	Ongoing
M	61	Cytological	Cell block	Lung	ADC	1–49%	22C3	WT	July 2018	0	Cisplatino + Pemetrexed	July 2018	September 2018
F	56	Histological	Biopsy	Lung	NSCLC favor ADC	1–49%	22C3	*KRAS* exon 2 p.G13C	December 2018	2	Carboplatin + Pemetrexed	January 2019	August 2019
M	71	Histological	Biopsy	Lung	NSCLC favor ADC	1–49%	SP263	*KRAS* exon 2 p.G12V	May 2019	1	Cisplatin + Pemetrexed	May 2019	July 2019
M	48	Histological	Biopsy	Pleura	NSCLC favor ADC	1–49%	SP263	WT	June 2019	1	Cisplatin + Pemetrexed	June 2019	October 2019
F	67	Cytological	Cell block	Lymphnode	NSCLC favor ADC	1–49%	SP263	WT	December 2019	2	Carboplatin + Pemetrexed	January 2020	January 2020
M	70	Cytological	Cell block	Lymphnode	NSCLC favor ADC	1–49%	SP263	*KRAS* exon 2 p.G12V	June 2021	1	Carboplatin + Gemcitabina	July 2021	September 2021
F	74	Cytological	Cell block	Lung	ADC	1–49%	SP263	*KRAS* exon 2 p.G12C	September 2020	1	Pemetrexed + Pembrolizumab	October 2020	October 2020
M	70	Histological	Biopsy	Lung	NSCLC favor ADC	≥50%	SP263	WT	June 2021	3	Supportive care	-	-
M	77	Cytological	Cell block	Lung	NSCLC favor SqCC	1–49%	SP263	WT	April 2021	2	Atezolizumab	May 2021	August 2021
F	71	Cytological	Cell block	Lymphnode	NSCLC favor ADC	≥50%	SP263	*KRAS* exon 2 p.G12A	February 2021	1	Pembrolizumab	March 2021	Ongoing
F	76	Histological	Biopsy	Lung	SqCC	1–49%	22C3	WT	August 2018	1	Nivolumab	January 2019	January 2019
M	72	Cytological	Cell block	Lymphnode	NSCLC favor ADC	1–49%	22C3	WT	October 2017	1	Cisplatin + Pemetrexed	October 2017	November 2017
M	46	Histological	Biopsy	Brain	NSCLC favor ADC	1–49%	SP263	WT	May 2019	0	Cisplatin + Pemetrexed	June 2019	January 2020
M	73	Histological	Resection	Lung	ADC	1–49%	SP263	WT	June 2020	0	Carboplatin + Pemetrexed	August 2020	October 2020
M	59	Cytological	Cell block	Lung	NSCLC favor ADC	≥50%	SP263	WT	July 2020	0	Pembrolizumab	August 2020	Ongoing
F	64	Histological	Biopsy	Lung	ADC	1–49%	SP263	WT	September 2020	1	Carboplatin + Pemetrexed + Pembrolizumab	October 2020	November 2020
M	75	Histological	Biopsy	Liver	ADC	1–49%	SP263	*EGFR* exon 19 p.E746_A750del	January 2021	1	Osimertinib	January 2021	Ongoing
M	68	Histological	Biopsy	Lung	ADC	≥50%	SP263	*KRAS* exon 2 p.G12C	December 2020	1	Pembrolizumab	December 2020	Ongoing

Abbreviations: ADC: adenocarcinoma; *ALK*: Anaplastic Lymphoma Receptor Tyrosine Kinase; *BRAF*: V-Raf Murine Sarcoma Viral Oncogene Homolog B1; *EGFR*: Epidermal Growth Factor Receptor; F: female; *KRAS*: Kirsten Rat Sarcoma Viral Oncogene Homolog; M: male; NOS: not otherwise specified; NSCLC: non-small cell lung cancer; WT: wild type; y: years.

## Data Availability

The data presented in this study are available on request from the corresponding author.

## Supplementary Materials

The supporting information can be downloaded at: https://www.mdpi.com/article/10.3390/ijms23158541/s1.
