# Supplementary material for: Evaluation of the Molecular Landscape in PD-L1 Positive Metastatic NSCLC: Data from Campania, Italy
Source: Int J Mol Sci. 2022 Aug 1;23(15):8541. doi: 10.3390/ijms23158541 (PMC9369105; doi:10.3390/ijms23158541)
Supplement: Supplementary file 1 [file ijms-23-08541-s001.zip › ijms-1819590-supplementary.pdf]

Table S1 Patients and samples characteristics with PD-L1 and molecular data.

Patient	Sex	Age	Sample type	Sample subtype	Site	Diagnosis	PD-L1	clone
1	M	64	Cytological	Cell block	Lung	NSCLC-NOS	1-50%	22C3
2	M	63	Histological	Biopsy	Lung	NSCLC favor ADC	>50%	22C3
3	M	68	Histological	Biopsy	Liver	ADC	1-50%	22C3
4	M	52	Histological	Resection	Brain	ADC	1-50%	22C3
5	F	61	Histological	Resection	Lung	NSCLC favor ADC	>50%	22C3
6	F	83	Histological	Biopsy	Lung	NSCLC favor ADC	1-50%	22C3
7	F	65	Cytological	Cell block	Lung	NSCLC favor ADC	1-50%	22C3
8	M	78	Histological	Biopsy	Lymphnode	NSCLC-NOS	>50%	22C3
9	F	81	Histological	Biopsy	Lung	NSCLC-NOS	1-50%	22C3
10	M	49	Histological	Biopsy	Lung	NSCLC favor ADC	1-50%	22C3
11	M	61	Cytological	Cell block	Lung	ADC	1-50%	22C3
12	F	76	Histological	Biopsy	Lung	SqCC	1-50%	22C3
13	F	63	Histological	Resection	Lung	ADC	>50%	22C3
14	M	72	Cytological	Cell block	Lung	NSCLC favor ADC	1-50%	22C3
15	M	73	Cytological	Cell block	Lymphnode	NSCLC favor ADC	>50%	22C3
16	F	78	Histological	Resection	Bone	SqCC	>50%	22C3
17	M	76	Histological	Biopsy	Lung	ADC	1-50%	22C3
18	M	52	Histological	Biopsy	Lung	ADC	1-50%	22C3
19	F	58	Cytological	Cell block	Lung	NSCLC-NOS	>50%	22C3
20	F	68	Histological	Biopsy	Lymphnode	NSCLC-NOS	>50%	22C3
21	M	70	Cytological	Cell block	Lymphnode	NSCLC favor ADC	>50%	22C3
22	F	78	Histological	Resection	Colon	NSCLC-NOS	1-50%	22C3
23	M	72	Cytological	Cell block	Lymphnode	NSCLC favor ADC	1-50%	22C3

24	M	62	Cytological	Cell block	Lymphnode	NSCLC favor ADC	>50%	22C3
25	F	60	Histological	Biopsy	Brain	NSCLC favor ADC	>50%	22C3
26	M	43	Histological	Resection	Brain	NSCLC favor ADC	1-50%	22C3
27	F	70	Cytological	Cell block	Lung	ADC	>50%	22C3
28	M	75	Histological	Biopsy	Lung	NSCLC favor ADC	>50%	22C3
29	F	76	Histological	Biopsy	Lung	NSCLC favor ADC	>50%	22C3
30	F	56	Histological	Biopsy	Lung	NSCLC favor ADC	1-50%	22C3
31	F	70	Cytological	Cell block	Lung	ADC	>50%	22C3
32	M	67	Histological	Biopsy	Pleura	NSCLC favor ADC	1-50%	22C3
33	M	59	Histological	Resection	Brain	NSCLC-NOS	1-50%	SP263
34	M	62	Histological	Resection	Lymphnode	ADC	>50%	22C3
35	F	58	Cytological	Cell block	Lymphnode	ADC	>50%	SP263
36	M	44	Cytological	Cell block	Lung	NSCLC-NOS	1-50%	SP263
37	M	60	Histological	Biopsy	Lung	NSCLC-NOS	1-50%	SP263
38	F	62	Histological	Biopsy	Bone	ADC	1-50%	SP263
39	M	50	Histological	Biopsy	Lung	NSCLC favor ADC	1-50%	SP263
40	M	68	Histological	Biopsy	Lung	NSCLC-NOS	>50%	SP263
41	M	64	Histological	Biopsy	Lung	NSCLC favor ADC	1-50%	SP263
42	F	50	Histological	Resection	Lung	ADC	1-50%	SP263
43	M	70	Histological	Biopsy	Brain	NSCLC favor ADC	>50%	SP263
44	M	53	Cytological	Cell block	Lung	NSCLC favor ADC	1-50%	SP263
45	M	62	Cytological	Cell block	Lymphnode	NSCLC favor ADC	>50%	SP263

46	F	68	Histological	Resection	Lymphnode	ADC	1-50%	SP263
47	M	65	Histological	Biopsy	Lung	ADC	>50%	SP263
48	M	78	Cytological	Smear	Lung	ADC	>50%	SP263
49	M	71	Histological	Biopsy	Lung	NSCLC favor ADC	1-50%	SP263
50	M	79	Histological	Biopsy	Lung	SqCC	1-50%	SP263
51	M	72	Cytological	Cell block	Lung	NSCLC favor ADC	1-50%	SP263
52	M	46	Histological	Biopsy	Brain	NSCLC favor ADC	1-50%	SP263
53	F	60	Histological	Biopsy	Lung	ADC	>50%	SP263
54	F	73	Histological	Biopsy	Lung	NSCLC favor ADC	>50%	SP263
55	F	49	Cytological	Cell block	Lymphnode	NSCLC favor ADC	>50%	SP263
56	M	85	Histological	Biopsy	Lung	NSCLC favor ADC	>50%	SP263
57	F	79	Histological	Biopsy	Brain	NSCLC-NOS	1-50%	SP263
58	M	48	Histological	Biopsy	Pleura	NSCLC favor ADC	1-50%	SP263
59	F	60	Histological	Biopsy	Lung	NSCLC favor ADC	>50%	SP263
60	M	68	Cytological	Cell block	Lung	SqCC	1-50%	SP263
61	M	65	Cytological	Cell block	Bone	ADC	1-50%	SP263
62	M	73	Histological	Biopsy	Lung	NSCLC favor SqCC	>50%	SP263
63	F	66	Histological	Biopsy	Lung	SqCC	1-50%	SP263
64	M	73	Cytological	Cell block	Lung	NSCLC favor SqCC	>50%	SP263
65	M	77	Histological	Biopsy	Lung	NSCLC favor ADC	>50%	SP263
66	M	77	Cytological	Cell block	Lung	NSCLC-NOS	>50%	SP263
67	F	62	Histological	Resection	Lymphnode	NSCLC favor ADC	>50%	SP263

68	M	65	Histological	Resection	Lymphnode	ADC	1-50%	SP263
69	M	65	Histological	Biopsy	Lymphnode	NSCLC-NOS	>50%	SP263
70	M	68	Histological	Biopsy	Lung	NSCLC favor ADC	>50%	SP263
71	M	79	Histological	Biopsy	Lung	ADC	1-50%	SP263
72	M	44	Histological	Biopsy	Lung	NSCLC-NOS	>50%	SP263
73	M	80	Cytological	Cell block	Lymphnode	NSCLC-NOS	>50%	SP263
74	F	66	Histological	Biopsy	Lung	NSCLC favor ADC	>50%	SP263
75	M	75	Cytological	Smear	Lung	ADC	1-50%	SP263
76	M	80	Histological	Resection	Brain	ADC	1-50%	SP263
77	M	49	Cytological	Cell block	Lung	NSCLC favor ADC	>50%	SP263
78	M	63	Cytological	Cell block	Lung	NSCLC favor ADC	>50%	SP263
79	F	67	Cytological	Cell block	Lymphnode	NSCLC favor ADC	1-50%	SP263
80	F	45	Histological	Resection	Lung	ADC	1-50%	SP263
81	F	61	Histological	Biopsy	Soft tissue	NSCLC favor ADC	>50%	SP263
82	M	72	Histological	Biopsy	Brain	ADC	1-50%	SP263
83	F	69	Cytological	Cell block	Lung	ADC	1-50%	SP263
84	M	68	Histological	Biopsy	Lymphnode	NSCLC-NOS	>50%	SP263
85	M	81	Histological	Biopsy	Lung	NSCLC favor ADC	1-50%	SP263
86	M	84	Cytological	Cell block	Lymphnode	ADC	1-50%	SP263
87	F	69	Cytological	Cell block	Soft tissue	ADC	1-50%	SP263
88	M	59	Histological	Biopsy	Lung	NSCLC favor ADC	>50%	SP263
89	F	76	Cytological	Cell block	Lung	ADC	1-50%	SP263
90	M	65	Cytological	Cell block	Lung	ADC	1-50%	SP263
91	M	56	Histological	Biopsy	Lung	NSCLC-NOS	>50%	SP263

92	M	57	Histological	Biopsy	Lung	NSCLC-NOS	>50%	SP263
93	M	70	Cytological	Cell block	Lymphnode	NSCLC favor ADC	1-50%	SP263
94	M	78	Histological	Biopsy	Lung	NSCLC favor ADC	>50%	SP263
95	F	50	Histological	Biopsy	Lung	NSCLC favor SqCC	>50%	SP263
96	M	74	Cytological	Cell block	Pleura	ADC	>50%	SP263
97	F	66	Histological	Resection	Brain	ADC	>50%	SP263
98	M	72	Cytological	Cell block	Lung	NSCLC favor ADC	1-50%	SP263
99	M	61	Histological	Biopsy	Pleura	NSCLC favor ADC	>50%	SP263
100	M	74	Histological	Biopsy	Lymphnode	ADC	>50%	SP263
101	M	77	Histological	Biopsy	Lymphnode	NSCLC favor ADC	1-50%	SP263
102	M	75	Histological	Biopsy	Lymphnode	ADC	>50%	SP263
103	M	78	Histological	Biopsy	Lymphnode	NSCLC favor ADC	>50%	SP263
104	F	63	Histological	Resection	Lung	ADC	1-50%	SP263
105	F	75	Histological	Biopsy	Lung	ADC	1-50%	SP263
106	M	57	Histological	Biopsy	Lung	ADC-SqCC	>50%	SP263
107	F	59	Cytological	Cell block	Adrenal gland	ADC	1-50%	SP263
108	M	77	Histological	Resection	Lung	ADC	>50%	SP263
109	M	64	Histological	Biopsy	Bone	ADC	>50%	SP263
110	F	76	Histological	Biopsy	Bone	NSCLC-NOS	>50%	SP263
111	M	73	Histological	Resection	Lung	ADC	1-50%	SP263
112	M	53	Histological	Biopsy	Lung	ADC-SqCC	1-50%	SP263
113	M	80	Cytological	Cell block	Lung	ADC	>50%	SP263
114	M	59	Cytological	Cell block	Lung	NSCLC favor ADC	>50%	SP263
115	F	74	Cytological	Cell block	Lung	ADC	1-50%	SP263

116	M	53	Cytological	Smear	Lung	ADC	1-50%	SP263
117	M	57	Histological	Resection	Lung	NSCLC-NOS	>50%	SP263
118	M	80	Cytological	Cell block	Lung	NSCLC favor ADC	1-50%	SP263
119	F	69	Histological	Biopsy	Lung	NSCLC favor ADC	1-50%	SP263
120	F	64	Histological	Biopsy	Lung	ADC	1-50%	SP263
121	M	76	Histological	Biopsy	Lung	ADC-SqCC	>50%	SP263
122	F	73	Histological	Biopsy	Lung	NSCLC favor ADC	>50%	SP263
123	M	47	Histological	Biopsy	Brain	NSCLC-NOS	1-50%	SP263
124	M	74	Cytological	Cell block	Lung	ADC	1-50%	SP263
125	M	52	Histological	Biopsy	Skin	ADC	1-50%	SP263
126	F	62	Cytological	Cell block	Lung	ADC	1-50%	SP263
127	M	63	Cytological	Cell block	Lung	NSCLC favor ADC	>50%	SP263
128	M	72	Cytological	Cell block	Lung	ADC	1-50%	SP263
129	M	78	Cytological	Cell block	Lung	SqCC	1-50%	SP263
130	M	70	Histological	Biopsy	Lung	NSCLC favor ADC	>50%	SP263
131	M	73	Cytological	Cell block	Lung	ADC	1-50%	SP263
132	M	75	Cytological	Cell block	Lung	NSCLC favor ADC	1-50%	SP263
133	M	93	Cytological	Cell block	Pleura	ADC	>50%	SP263
134	M	62	Cytological	Smear	Pleura	ADC	>50%	SP263
135	F	76	Cytological	Smear	Lung	NSCLC-NOS	>50%	SP263
136	F	55	Cytological	Cell block	Lung	NSCLC-NOS	>50%	SP263
137	F	71	Histological	Biopsy	Lung	ADC	1-50%	SP263
138	M	74	Histological	Biopsy	Adrenal gland	NSCLC-NOS	>50%	SP263
139	M	77	Cytological	Cell block	Lung	NSCLC favor SqCC	1-50%	SP263
140	M	75	Histological	Biopsy	Liver	ADC	1-50%	SP263

141	M	68	Histological	Biopsy	Lung	ADC	>50%	SP263
142	F	69	Histological	Biopsy	Lung	NSCLC favor ADC	>50%	SP263
143	F	92	Histological	Biopsy	Pleura	ADC	1-50%	SP263
144	F	67	Cytological	Cell block	Lymphnode	ADC	1-50%	SP263
145	F	71	Cytological	Cell block	Lymphnode	NSCLC favor ADC	>50%	SP263
146	F	80	Cytological	Cell block	Lung	NSCLC favor ADC	>50%	SP263
147	F	62	Histological	Biopsy	Brain	NSCLC-NOS	1-50%	SP263
148	F	54	Histological	Resection	Lung	ADC	1-50%	SP263
149	M	64	Histological	Biopsy	Lung	NSCLC favor ADC	1-50%	SP263
150	F	72	Cytological	Cell block	Lung	NSCLC favor ADC	>50%	SP263
151	F	69	Histological	Biopsy	Lung	ADC	1-50%	SP263
152	M	72	Histological	Biopsy	Lung	SqCC	1-50%	SP263
153	F	59	Histological	Resection	Lung	NSCLC-NOS	>50%	SP263
154	F	85	Histological	Biopsy	Lung	NSCLC-NOS	1-50%	SP263
155	F	74	Histological	Resection	Brain	ADC	>50%	SP263
156	M	76	Histological	Biopsy	Lung	NSCLC-NOS	1-50%	SP263
157	F	75	Histological	Resection	Lung	ADC	>50%	SP263
158	M	78	Histological	Biopsy	Lung	SqCC	>50%	SP263
159	M	77	Histological	Biopsy	Bone	NSCLC-NOS	>50%	SP263
160	F	58	Histological	Resection	Brain	ADC	1-50%	SP263
161	M	61	Histological	Biopsy	Adrenal gland	NSCLC-NOS	>50%	SP263
162	M	61	Histological	Biopsy	Lung	NSCLC-NOS	>50%	SP263
163	M	62	Histological	Biopsy	Pleura	ADC	1-50%	SP263
164	M	77	Histological	Biopsy	Liver	ADC	1-50%	SP263
165	F	75	Histological	Biopsy	Soft tissue	NSCLC-NOS	>50%	SP263
166	F	72	Histological	Biopsy	Liver	ADC	>50%	SP263

167	F	53	Histological	Biopsy	Lung	NSCLC favor ADC	>50%	SP263

Platform	Molecular platform	EGFR	KRAS	NRAS	BRAF	KIT	PDGFRA	PIK3CA
Ventana-Roche	NGS	WT	WT	WT	WT	WT	WT	WT
Ventana-Roche	NGS	WT	p.G12C	WT	WT	WT	WT	WT
Ventana-Roche	NGS	WT	WT	WT	WT	WT	WT	WT
Ventana-Roche	NGS	WT	p.G12V	WT	WT	WT	WT	WT
Ventana-Roche	NGS	WT	p.G12C	WT	WT	WT	WT	WT
Ventana-Roche	NGS	WT	WT	WT	WT	WT	WT	WT
Ventana-Roche	NGS	WT	WT	WT	WT	WT	WT	WT
Ventana-Roche	NGS	WT	WT	WT	WT	WT	WT	WT
Ventana-Roche	NGS	WT	p.G12R	WT	WT	WT	WT	WT
Ventana-Roche	NGS	WT	WT	WT	WT	WT	WT	WT
Ventana-Roche	NGS	WT	WT	WT	WT	WT	WT	WT
Ventana-Roche	NGS	WT	WT	WT	WT	WT	WT	WT
Ventana-Roche	NGS	WT	p.G12C	WT	WT	WT	WT	WT
Ventana-Roche	NGS	WT	WT	WT	WT	WT	WT	WT
Ventana-Roche	NGS	WT	WT	WT	WT	WT	WT	WT
Ventana-Roche	NGS	WT	WT	WT	WT	WT	WT	WT
Ventana-Roche	RT-qPCR	p.S768I	WT	---	WT	---	---	---
Ventana-Roche	NGS	WT	WT	WT	WT	WT	WT	p.E542K
Ventana-Roche	NGS	p.L858R	WT	WT	WT	WT	WT	WT
Ventana-Roche	NGS	WT	WT	WT	WT	WT	WT	WT
Ventana-Roche	NGS	WT	WT	WT	p.V600E	WT	WT	WT
Ventana-Roche	NGS	WT	WT	WT	WT	WT	WT	WT
Ventana-Roche	NGS	WT	WT	WT	WT	WT	WT	WT

Ventana-Roche	NGS	WT	WT	WT	WT	WT	WT	WT
Ventana-Roche	NGS	WT	p.G12A	WT	WT	WT	WT	WT
Ventana-Roche	NGS	WT	WT	WT	WT	WT	WT	WT
Ventana-Roche	NGS	WT	WT	WT	WT	WT	WT	WT
Ventana-Roche	NGS	WT	WT	WT	WT	WT	WT	WT
Ventana-Roche	NGS	WT	p.G12C	WT	WT	WT	WT	WT
Ventana-Roche	NGS	WT	p.G13C	WT	WT	WT	WT	WT
Ventana-Roche	NGS	WT	p.Q61H	WT	WT	WT	WT	WT
Ventana-Roche	NGS	p.E746_A750del	WT	WT	WT	WT	WT	WT
Ventana-Roche	NGS	WT	WT	WT	WT	WT	WT	WT
Ventana-Roche	NGS	WT	WT	WT	WT	WT	WT	WT
Ventana-Roche	NGS	WT	WT	WT	WT	WT	WT	WT
Ventana-Roche	NGS	WT	WT	p.G12D	WT	WT	WT	WT
Ventana-Roche	NGS	WT	WT	WT	WT	WT	WT	WT
Ventana-Roche	NGS	WT	p.G12C	WT	WT	WT	WT	WT
Ventana-Roche	NGS	WT	WT	WT	WT	WT	WT	WT
Ventana-Roche	NGS	WT	WT	WT	WT	WT	WT	WT
Ventana-Roche	NGS	WT	WT	WT	WT	WT	WT	WT
Ventana-Roche	NGS	WT	WT	WT	WT	WT	WT	WT
Ventana-Roche	NGS	WT	p.G12C	WT	WT	WT	WT	WT
Ventana-Roche	NGS	WT	p.G13C	WT	WT	WT	WT	WT
Ventana-Roche	NGS	WT	WT	WT	WT	WT	WT	WT

Ventana-Roche	NGS	WT	WT	WT	WT	WT	WT	WT
Ventana-Roche	NGS	WT	WT	WT	WT	WT	WT	WT
Ventana-Roche	NGS	WT	WT	WT	WT	WT	WT	WT
Ventana-Roche	NGS	WT	p.G12V	WT	WT	WT	WT	WT
Ventana-Roche	NGS	WT	WT	WT	WT	WT	WT	WT
Ventana-Roche	NGS	WT	p.G12C	WT	WT	WT	WT	WT
Ventana-Roche	NGS	WT	WT	WT	WT	WT	WT	WT
Ventana-Roche	NGS	WT	p.G13D	WT	WT	WT	WT	WT
Ventana-Roche	NGS	WT	p.G12V	WT	WT	WT	WT	WT
Ventana-Roche	NGS	WT	WT	WT	WT	WT	WT	WT
Ventana-Roche	NGS	WT	p.G12D	WT	WT	WT	WT	WT
Ventana-Roche	NGS	p.L858R	WT	WT	WT	WT	WT	WT
Ventana-Roche	NGS	WT	WT	WT	WT	WT	WT	WT
Ventana-Roche	NGS	WT	WT	WT	p.G466V	WT	WT	WT
Ventana-Roche	NGS	WT	WT	WT	WT	WT	WT	WT
Ventana-Roche	NGS	p.G719A + p.T790M	WT	WT	WT	WT	WT	WT
Ventana-Roche	NGS	WT	WT	WT	WT	WT	WT	WT
Ventana-Roche	NGS	WT	WT	WT	WT	WT	WT	WT
Ventana-Roche	NGS	WT	WT	WT	WT	WT	WT	WT
Ventana-Roche	NGS	WT	WT	WT	WT	WT	WT	WT
Ventana-Roche	NGS	WT	WT	WT	WT	WT	WT	WT
Ventana-Roche	NGS	WT	WT	WT	WT	WT	WT	WT

Ventana-Roche	NGS	WT	p.G12A	WT	WT	WT	WT	WT
Ventana-Roche	NGS	WT	WT	WT	WT	WT	WT	WT
Ventana-Roche	NGS	WT	WT	WT	WT	WT	WT	WT
Ventana-Roche	NGS	WT	WT	WT	WT	WT	WT	WT
Ventana-Roche	NGS	p.LE746_S752del	WT	WT	WT	WT	WT	WT
Ventana-Roche	NGS	WT	WT	WT	WT	WT	WT	WT
Ventana-Roche	NGS	WT	p.G12V	WT	WT	WT	WT	WT
Ventana-Roche	NGS	WT	p.G12C	WT	WT	WT	WT	WT
Ventana-Roche	NGS	WT	p.G12C	WT	WT	WT	WT	WT
Ventana-Roche	NGS	WT	WT	WT	WT	WT	WT	WT
Ventana-Roche	NGS	p.L858R	WT	WT	WT	WT	WT	WT
Ventana-Roche	NGS	WT	WT	WT	WT	WT	WT	WT
Ventana-Roche	NGS	p.L858R	WT	WT	WT	WT	WT	WT
Ventana-Roche	NGS	WT	p.G12V	WT	WT	WT	WT	WT
Ventana-Roche	NGS	WT	p.Q61H	WT	WT	WT	WT	WT
Ventana-Roche	NGS	WT	p.G12C	WT	WT	WT	WT	WT
Ventana-Roche	NGS	p.E746_A750del	WT	WT	WT	WT	WT	WT
Ventana-Roche	NGS	WT	p.G12V	WT	WT	WT	WT	WT
Ventana-Roche	NGS	WT	p.G12C	WT	WT	WT	WT	WT
Ventana-Roche	NGS	p.S768_D760dup	WT	WT	WT	WT	WT	WT
Ventana-Roche	NGS	WT	p.Q61H	WT	WT	WT	WT	WT
Ventana-Roche	NGS	WT	WT	WT	WT	WT	WT	WT
Ventana-Roche	NGS	WT	WT	WT	WT	WT	WT	WT
Ventana-Roche	NGS	WT	p.G12C	WT	WT	WT	WT	WT

Ventana-Roche	NGS	WT	p.G12C	WT	WT	WT	WT	WT
Ventana-Roche	NGS	WT	p.G12V	WT	WT	WT	WT	WT
Ventana-Roche	NGS	WT	p.G12C	WT	WT	WT	WT	WT
Ventana-Roche	NGS	p.I744_K745insKIPVAI	WT	WT	WT	WT	WT	WT
Ventana-Roche	NGS	WT	p.G12A	WT	WT	WT	WT	WT
Ventana-Roche	NGS	WT	WT	WT	WT	WT	WT	WT
Ventana-Roche	NGS	WT	WT	WT	WT	WT	WT	WT
Ventana-Roche	NGS	WT	p.G12C	WT	WT	WT	WT	WT
Ventana-Roche	NGS	WT	p.G12D	WT	WT	WT	WT	WT
Ventana-Roche	NGS	WT	p.G12V	WT	WT	WT	WT	WT
Ventana-Roche	NGS	WT	WT	WT	WT	WT	WT	WT
Ventana-Roche	NGS	WT	WT	WT	WT	WT	WT	WT
Ventana-Roche	NGS	WT	WT	WT	WT	WT	WT	WT
Ventana-Roche	NGS	WT	p.G12C	WT	WT	WT	WT	WT
Ventana-Roche	NGS	WT	WT	WT	WT	WT	WT	WT
Ventana-Roche	NGS	WT	WT	WT	WT	WT	WT	WT
Ventana-Roche	NGS	WT	p.G12C	WT	WT	WT	WT	WT
Ventana-Roche	NGS	WT	p.G12V	WT	WT	WT	WT	WT
Ventana-Roche	NGS	WT	WT	WT	WT	WT	WT	WT
Ventana-Roche	NGS	WT	WT	WT	WT	WT	WT	WT
Ventana-Roche	NGS	p.E746_A750del	WT	WT	WT	WT	WT	WT
Ventana-Roche	NGS	WT	p.G12C	WT	WT	WT	WT	WT
Ventana-Roche	NGS	WT	WT	WT	WT	WT	WT	WT
Ventana-Roche	NGS	WT	p.G12C	WT	WT	WT	WT	WT

Ventana-Roche	NGS	WT	p.G12C	WT	WT	WT	WT	WT
Ventana-Roche	NGS	WT	WT	WT	WT	WT	WT	p.E545K
Ventana-Roche	NGS	WT	WT	WT	WT	WT	WT	WT
Ventana-Roche	NGS	WT	WT	WT	p.G469A	WT	WT	WT
Ventana-Roche	NGS	WT	WT	WT	WT	WT	WT	WT
Ventana-Roche	NGS	WT	WT	WT	WT	WT	WT	WT
Ventana-Roche	NGS	p.L858R	WT	WT	WT	WT	WT	WT
Ventana-Roche	NGS	WT	WT	WT	WT	WT	WT	WT
Ventana-Roche	NGS	WT	p.G12V	WT	WT	WT	WT	WT
Ventana-Roche	NGS	WT	WT	WT	WT	WT	WT	WT
Ventana-Roche	NGS	WT	WT	WT	WT	WT	WT	WT
Ventana-Roche	NGS	WT	p.G12C	WT	WT	WT	WT	WT
Ventana-Roche	NGS	WT	WT	WT	WT	WT	WT	WT
Ventana-Roche	NGS	WT	WT	WT	WT	WT	WT	WT
Ventana-Roche	NGS	WT	WT	WT	WT	WT	WT	WT
Ventana-Roche	NGS	WT	WT	WT	WT	WT	WT	WT
Ventana-Roche	NGS	WT	WT	WT	WT	WT	WT	WT
Ventana-Roche	NGS	WT	p.G12C	WT	WT	WT	WT	WT
Ventana-Roche	RT-qPCR	WT	WT	---	p.V600E	---	---	---
Ventana-Roche	NGS	WT	WT	WT	WT	WT	WT	WT
Ventana-Roche	NGS	WT	WT	WT	WT	WT	WT	WT
Ventana-Roche	NGS	p.L858R	WT	WT	WT	WT	WT	WT
Ventana-Roche	NGS	WT	p.G12V	WT	WT	WT	WT	WT
Ventana-Roche	NGS	WT	WT	WT	WT	WT	WT	WT
Ventana-Roche	NGS	p.E746_A750del	WT	WT	WT	WT	WT	WT

Ventana-Roche	NGS	WT	p.G12C	WT	WT	WT	WT	WT
Ventana-Roche	NGS	WT	WT	WT	WT	WT	WT	WT
Ventana-Roche	NGS	WT	p.G12D	WT	WT	WT	WT	WT
Ventana-Roche	NGS	WT	p.G12V	WT	WT	WT	WT	WT
Ventana-Roche	NGS	WT	p.G12A	WT	WT	WT	WT	WT
Ventana-Roche	NGS	p.L858R	WT	WT	WT	WT	WT	WT
Ventana-Roche	NGS	p.E746_A750del	WT	WT	WT	WT	WT	WT
Ventana-Roche	NGS	WT	WT	WT	WT	WT	WT	WT
Ventana-Roche	NGS	WT	p.G13R	WT	WT	WT	WT	WT
Ventana-Roche	NGS	p.L858R	WT	WT	WT	WT	WT	WT
Ventana-Roche	NGS	p.L858R	WT	WT	WT	WT	WT	WT
Ventana-Roche	NGS	WT	WT	WT	WT	WT	WT	WT
Ventana-Roche	NGS	WT	WT	WT	WT	WT	WT	WT
Ventana-Roche	NGS	p.E709_T710insD	WT	WT	WT	WT	WT	WT
Ventana-Roche	NGS	p.E746_A750del	WT	WT	WT	WT	WT	WT
Ventana-Roche	NGS	WT	p.G12C	WT	WT	WT	WT	WT
Ventana-Roche	NGS	WT	WT	WT	WT	WT	WT	WT
Ventana-Roche	NGS	WT	WT	WT	WT	WT	WT	WT
Ventana-Roche	NGS	WT	p.G12C	WT	WT	WT	WT	p.E545K
Ventana-Roche	NGS	WT	WT	WT	WT	WT	WT	WT
Ventana-Roche	NGS	WT	WT	WT	WT	WT	WT	WT
Ventana-Roche	NGS	WT	p.G12C	WT	WT	WT	WT	WT
Ventana-Roche	NGS	WT	WT	WT	WT	WT	WT	WT
Ventana-Roche	NGS	WT	p.G12V	WT	WT	WT	WT	WT
Ventana-Roche	NGS	WT	p.G12C	WT	WT	WT	WT	WT
Ventana-Roche	RT-qPCR	WT	p.G12A	---	WT	---	---	---

Ventana-Roche	NGS	WT	WT	WT	WT	WT	WT	WT

ALK	clone	ROS1	clone
Negative	D5F3	Negative	D4D6
Negative	D5F3	Negative	D4D6
Negative	D5F3	Negative	D4D6
Negative	D5F3	Negative	D4D6
Negative	D5F3	Negative	D4D6
Negative	D5F3	Negative	D4D6
Negative	D5F3	Negative	D4D6
Negative	D5F3	Negative	D4D6
Negative	D5F3	Negative	D4D6
Negative	D5F3	Negative	D4D6
Negative	D5F3	Negative	D4D6
Negative	D5F3	Negative	D4D6
Negative	D5F3	Negative	D4D6
Negative	D5F3	Negative	D4D6
Negative	D5F3	Negative	D4D6
Negative	D5F3	Negative	D4D6
Negative	D5F3	Negative	D4D6
Negative	D5F3	Negative	D4D6
Negative	D5F3	Negative	D4D6
Positive	D5F3	Negative	D4D6
Negative	D5F3	Negative	D4D6
Negative	D5F3	Negative	D4D6
Negative	D5F3	Negative	D4D6

Negative	D5F3	Negative	D4D6
Negative	D5F3	Negative	D4D6
Negative	D5F3	Negative	D4D6
Negative	D5F3	Negative	D4D6
Negative	D5F3	Negative	D4D6
Negative	D5F3	Negative	D4D6
Negative	D5F3	Negative	D4D6
Negative	D5F3	Negative	D4D6
Negative	D5F3	Negative	D4D6
Negative	D5F3	Negative	D4D6
Positive	D5F3	Negative	D4D6
Negative	D5F3	Negative	D4D6
Negative	D5F3	Negative	D4D6
Negative	D5F3	Negative	D4D6
Negative	D5F3	Negative	D4D6
Negative	D5F3	Negative	D4D6
Negative	D5F3	Negative	D4D6
Negative	D5F3	Negative	D4D6
Negative	D5F3	Negative	D4D6
Negative	D5F3	Negative	D4D6
Negative	D5F3	Negative	D4D6
Negative	D5F3	Negative	D4D6

Negative	D5F3	Negative	D4D6
Negative	D5F3	Negative	D4D6
Negative	D5F3	Negative	D4D6
Negative	D5F3	Negative	D4D6
Negative	D5F3	Negative	D4D6
Negative	D5F3	Negative	D4D6
Negative	D5F3	Negative	D4D6
Negative	D5F3	Negative	D4D6
Negative	D5F3	Negative	D4D6
Negative	D5F3	Negative	D4D6
Negative	D5F3	Negative	D4D6
Negative	D5F3	Negative	D4D6
Negative	D5F3	Negative	D4D6
Negative	D5F3	Negative	D4D6
Negative	D5F3	Negative	D4D6
Negative	D5F3	Negative	D4D6
Negative	D5F3	Negative	D4D6
Negative	D5F3	Negative	D4D6
Negative	D5F3	Negative	D4D6
Negative	D5F3	Negative	D4D6
Negative	D5F3	Negative	D4D6
Negative	D5F3	Negative	D4D6

Negative	D5F3	Negative	D4D6
Negative	D5F3	Negative	D4D6
Negative	D5F3	Negative	D4D6
Negative	D5F3	Negative	D4D6
Negative	D5F3	Negative	D4D6
Negative	D5F3	Negative	D4D6
Negative	D5F3	Negative	D4D6
Negative	D5F3	Negative	D4D6
Negative	D5F3	Negative	D4D6
Negative	D5F3	Negative	D4D6
Negative	D5F3	Negative	D4D6
Negative	D5F3	Negative	D4D6
Negative	D5F3	Negative	D4D6
Negative	D5F3	Negative	D4D6
Negative	D5F3	Negative	D4D6
Negative	D5F3	Negative	D4D6
Negative	D5F3	Negative	D4D6
Negative	D5F3	Negative	D4D6
Negative	D5F3	Negative	D4D6
Negative	D5F3	Negative	D4D6
Negative	D5F3	Negative	D4D6
Negative	D5F3	Negative	D4D6
Negative	D5F3	Negative	D4D6
Negative	D5F3	Negative	D4D6

Negative	D5F3	Negative	D4D6
Negative	D5F3	Negative	D4D6
Negative	D5F3	Negative	D4D6
Negative	D5F3	Negative	D4D6
Negative	D5F3	Negative	D4D6
Negative	D5F3	Negative	D4D6
Negative	D5F3	Negative	D4D6
Negative	D5F3	Negative	D4D6
Negative	D5F3	Negative	D4D6
Negative	D5F3	Negative	D4D6
Negative	D5F3	Negative	D4D6
Negative	D5F3	Negative	D4D6
Negative	D5F3	Negative	D4D6
Negative	D5F3	Negative	D4D6
Negative	D5F3	Negative	D4D6
Negative	D5F3	Negative	D4D6
Negative	D5F3	Negative	D4D6
Negative	D5F3	Negative	D4D6
Negative	D5F3	Negative	D4D6
Negative	D5F3	Negative	D4D6
Negative	D5F3	Negative	D4D6
Negative	D5F3	Negative	D4D6
Negative	D5F3	Negative	D4D6
Negative	D5F3	Negative	D4D6

Negative	D5F3	Negative	D4D6
Negative	D5F3	Negative	D4D6
Negative	D5F3	Negative	D4D6
Negative	D5F3	Negative	D4D6
Negative	D5F3	Negative	D4D6
Negative	D5F3	Negative	D4D6
Negative	D5F3	Negative	D4D6
Positive	D5F3	Negative	D4D6
Negative	D5F3	Negative	D4D6
Negative	D5F3	Negative	D4D6
Negative	D5F3	Negative	D4D6
Negative	D5F3	Negative	D4D6
Negative	D5F3	Negative	D4D6
Negative	D5F3	Negative	D4D6
Negative	D5F3	Negative	D4D6
Negative	D5F3	Negative	D4D6
Negative	D5F3	Negative	D4D6
Negative	D5F3	Negative	D4D6
Negative	D5F3	Negative	D4D6
EML4(13)-ALK(20)	---	WT	---
Negative	D5F3	Negative	D4D6
Negative	D5F3	Negative	D4D6
Negative	D5F3	Positive	D4D6
Negative	D5F3	Negative	D4D6
WT	---	WT	---

Negative	D5F3	Negative	D4D6
EML4(6)-ALK(20)	---	WT	---
WT	---	WT	---
WT	---	WT	---
WT	---	WT	---
Negative	D5F3	Negative	D4D6
Negative	D5F3	Negative	D4D6
unknown-ALK(20)	---	WT	---
Negative	D5F3	Negative	D4D6
Negative	D5F3	Negative	D4D6
Negative	D5F3	Negative	D4D6
Negative	D5F3	Negative	D4D6
Negative	D5F3	Negative	D4D6
WT	---	WT	---
WT	---	WT	---
Negative	D5F3	Negative	D4D6
WT	---	WT	---
WT	---	WT	---
WT	---	WT	---
WT	---	WT	---
WT	---	WT	---
WT	---	WT	---
Positive	D5F3	Negative	D4D6
Negative	D5F3	Negative	D4D6
Negative	D5F3	Negative	D4D6
Negative	D5F3	Negative	D4D6

Negative	D5F3	Negative	D4D6
